# Inorganic dietary nanoparticles in intestinal barrier function of inflammatory bowel disease: allies or adversaries?

**DOI:** 10.3389/fimmu.2025.1563504

**Published:** 2025-04-09

**Authors:** Duo Luo, Guifang Luo, Haoming Xu, Kangbao Li, Zhaotao Li, Cong Zhang

**Affiliations:** ^1^ Department of Geriatrics, Guangzhou First People’s Hospital, Guangzhou Medical University, Guangzhou, China; ^2^ Department of Gastroenterology and Hepatology, Guangzhou First People’s Hospital, Guangzhou Medical University, Guangzhou, China; ^3^ Department of Gastroenterology, The First People’s Hospital of Foshan, Foshan, China

**Keywords:** inorganic dietary nanoparticles, inflammatory bowel disease, intestinal barrier, immune barrier, biological barrier

## Abstract

Inorganic dietary nanoparticles (IDNPs) are frequently utilized as food additives and in packaging, resulting in their exposure becoming a substantial yet often overlooked concern for patients with inflammatory bowel disease (IBD). Considering that impaired intestinal barrier function plays a central role in the pathogenesis of IBD, this review concentrates on the roles and mechanisms of IDNPs in the intestinal barrier (physical, chemical, biological, and immune barriers) of IBD patients. Previous studies have shown that different types of nanoparticles have varying effects on animals in diverse states. In this context, factors such as the source, size, shape, dosage, and duration of action of the nanoparticles, as well as the species, gender, dietary habits, and age of the animals, significantly influence research outcomes. Future studies should undertake more comprehensive explorations into the effects and mechanisms of IDNPs with diverse sources and properties in IBD patients.

## 1 Introduction

Inorganic dietary nanoparticles (IDNPs) are defined as inorganic materials with dimensions typically ranging from 1 to 100 nanometers in food, exhibiting distinct physicochemical properties compared to their bulk counterparts. The IDNPs can be broadly classified into metal nanoparticles (e.g., gold, silver, zinc), metal oxide nanoparticles (e.g., titanium dioxide, iron oxide, zinc oxide), and composite nanoparticles (NPs) that combine organic and inorganic materials (1, 2). Their nanoscale dimension endows them with high surface area-to-volume ratios, which enhance their reactivity and interaction with biological molecules (3). Additionally, their size, shape, and surface properties can be tailored to influence their absorption, distribution, and bioavailability within the body (4). IDNPs are now widely utilized in various scenarios such as food additives and food packaging, and their safety and environmental toxicity are garnering increasing attention from the public.

Inflammatory bowel disease (IBD) is a chronic inflammatory disease primarily affecting the gastrointestinal tract, mainly encompassing Crohn’s disease (CD) and ulcerative colitis (UC). CD can manifest anywhere from the mouth to the anus, featuring transmural inflammation that may lead to complications such as strictures and fistulas (5). In contrast, UC is confined to the colonic mucosa and is characterized by continuous inflammation extending proximally from the rectum (5, 6). Clinically, IBD presents with diverse gastrointestinal symptoms, including abdominal pain, diarrhea, and weight loss, along with extraintestinal manifestations like arthritis and skin lesions (5). IBD is characterized by periods of exacerbation and remission, significantly impacting the morbidity and quality of life of patients. The management of IBD involves medication intervention, dietary adjustments, and surgical choices, but there is no single method that can completely cure patients (7). The etiology of IBD is multifactorial, involving genetic predisposition, environmental triggers, and deregulated immune responses to gut microbiota (8). As a type of environmental factor, the conclusions drawn from previous studies on the role of IDNPs in IBD are inconsistent (9, 10). Therefore, we have reviewed the mechanism of function of IDNPs in the intestinal barrier of IBD in order to provide insights for the future diagnosis, treatment, and prevention of the disease.

## 2 Inorganic dietary nanoparticles

### 2.1 The source of IDNPs

In modern life, IDNPs are widely utilized across various fields. Titanium dioxide nanoparticles (TiO_2_ NPs) are commonly used as a white pigment and brightening agent in refined foods, including confectionery items, white sauces, and icings (11–13). It is also found in toothpaste and nondairy creamers (14). Silicon dioxide nanoparticles (SiO_2_ NPs) are often added to powdered foods as an anticaking agent, including salt, icing sugar, spices, dried milk, and dry mixes (15, 16). Silver nanoparticles (Ag NPs) are used as a coloring agent for products like cakes, ice creams, frozen desserts, chocolates, and antibacterial food packaging (17). Iron oxide nanoparticles (Fe_2_O_3_ NPs) function as a food colorant as well as an ingredient in cosmetics and pharmaceutical coatings (18). Zinc oxide nanoparticles (ZnO NPs) can be found in dietary supplements and functional foods (19, 20). Moreover, TiO_2_ is used for pill coatings in the pharmaceutical industry, while AlSi is applied in paper manufacturing and powder fabrication.

### 2.2 The exposure levels of different IDNPs

In recent years, the exposure levels of TiO_2_ have been widely studied, and there are significant differences in exposure levels among different age groups in different countries due to varying dietary habits and the content of IDNPs in food. In China, children aged 2-5 are exposed to 0.337 mg TiO_2_/kgbw/day, whereas elderly individuals (aged 70 and above) are exposed to 0.061 mg TiO_2_/kgbw/day (21). In the Netherlands, the average exposure level among the population (aged 2 and above) ranges from 0.06 to 0.67 mg TiO_2_/kgbw/day (22). In the United States and the United Kingdom, children under 10 years old are exposed to 1-2 and 2-3 mg TiO_2_/kgbw/day, respectively; while the exposure levels for other age groups are 0.2-0.7 mg and 1 mg TiO_2_/kgbw/day, respectively (11). In Germany, children under 10 years old exhibit higher exposure levels compared to other countries, with those aged 3-9 exposed to 3.3 mg TiO_2_/kgbw/day (23). Research on the exposure levels of other IDNPs is relatively scarce. According to the European Food Safety Authority, infants are exposed to a daily dose of food-grade silica (E551) ranging from 0.8 to 74.2 mg/kgbw, children from 2.7 to 31.2 mg/kgbw, and adults from 0.9 to 13.2 mg/kgbw (24). The daily exposure level of silver (E174) for individuals ranging from children to adults is 0.03-2.6 mg/kgbw (24).

### 2.3 Intestinal absorption mode of IDNPs

IDNPs enter the digestive tract through the mouth along with food and, propelled by the peristalsis of the gastrointestinal tract, reach the small intestine. Some of them are absorbed into the system via the intestine, while the vast majority are excreted in the feces (25, 26). In experiments involving human volunteers, it was discovered that the blood TiO_2_ level began to rise 2 hours after oral ingestion, peaking between 8-12 hours later (27, 28). Furthermore, the presence of TiO_2_ nanoparticles was detected in the liver and spleen of both mice and humans, suggesting that IDNPs can accumulate within the body (29–31). The mechanisms by which IDNPs are absorbed into the systemic circulation from the intestine primarily encompass three categories: 1) trans-intestinal epithelial cell pathway; 2) transport via tight junctions adjacent to cells; 3) transport through transcytosis across M cells in Peyer’s patches (32, 33). The ability of IDNPs to penetrate the intestinal epithelium is dependent on their diameter (34). Specifically, IDNPs with diameters less than 150 µm are capable of penetrating the intestinal epithelium, whereas those with diameters under 2.5 µm are absorbed by M cells in Peyer’s patches (34, 35).

### 2.4 Factors affecting the function of IDNPs

The inherent characteristics of IDNPs, encompassing their composition, size, shape, surface properties, and aggregation state, can influence their functionality. IDNPs can consist of various inorganic substances, including titanium dioxide, silicon dioxide, zinc oxide, iron oxide, and more. Their size, ranging from a few nanometers to several hundred nanometers, and shape—whether sheet-like, spherical, cylindrical, or rectangular—are determined by their manufacturing method. Furthermore, their surface properties are dependent on the types of molecules present on their surface (36–38). Nevertheless, upon entering the digestive tract, the properties of these nanoparticles may undergo significant changes due to interactions with food ingredients or substances naturally present in the gastrointestinal tract (39).

**Figure 1:**
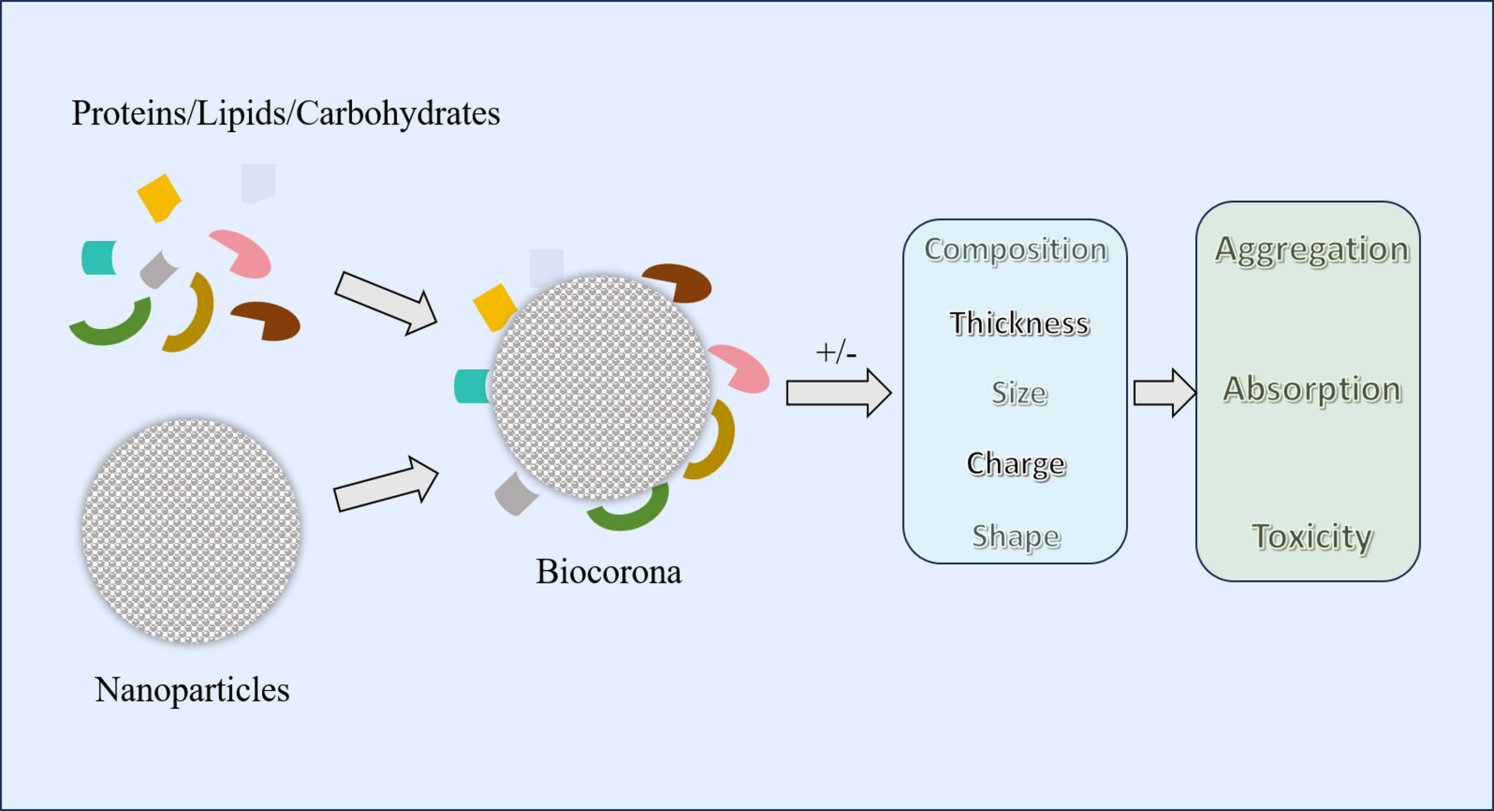
Formation of biocorona and its impact on different aspects of nanoparticles. Different food ingredients or inherent components of the gastrointestinal tract (proteins, lipids, carbohydrates, etc.) can form a biocorona with nanoparticles. The formation of a biocorona may alter the composition, size, thickness, charge, and shape of nanoparticles, thereby changing their aggregation, absorption, and toxicity.

After interacting with food ingredients or naturally biological substances in the gastrointestinal tract (proteins, lipids, carbohydrates, etc.), biological corona can form around the IDNPs (40, 41). The corona not only alter the structure and function of the ingredients themselves but also influence the absorption, accumulation, and toxicity of IDNPs (42, 43) (**Figure 1**). First, the adsorption of substances onto IDNPs can modify the composition, thickness, and charge of the surface layer, thereby affecting its aggregation state (44, 45). For instance, albumin can facilitate the aggregation of SiO_2_ NPs, possibly due to charge neutralization and bridging effects (40). Conversely, adding proteins to TiO_2_ and ZnO NPs can enhance their dispersion, possibly due to the formation of protein corona that increase the repulsion between particles (41). Additionally, the formation of biological corona can also impact the absorption of nanoparticles. Research has revealed that in the presence of proteins, the absorption of Fe_3_O_4_ NPs by Caco-2 cell monolayers increases, possibly due to the protein corona promoting the dispersion of nanoparticles (46). Finally, the formation of protein corona may alter the biocompatibility of nanoparticles, thereby affecting the toxicity of IDNPs. Study has found that the surface adsorption of bovine lactoferrin on silver nanoparticles reduces its cytotoxicity towards THP-1 cell lines (42). Therefore, the function of IDNPs in the body is a complex state that varies depending on the type of food consumed, the location in the intestine, and the disease status. Conversely, nanoparticles can also influence the activity of intrinsic biological proteins in the gastrointestinal tract while being influenced by the properties of food proteins. For example, TiO_2_ and SiO_2_ NPs can interact with trypsin and reduce its activity (47). Additionally, Ag NPs have been shown to diminish the activity of gastric protease due to surface denaturation caused by the adsorption of digestive enzymes onto the nanoparticles’ surface (48).

## 3 The pathogenesis of IBD

**Figure 2:**
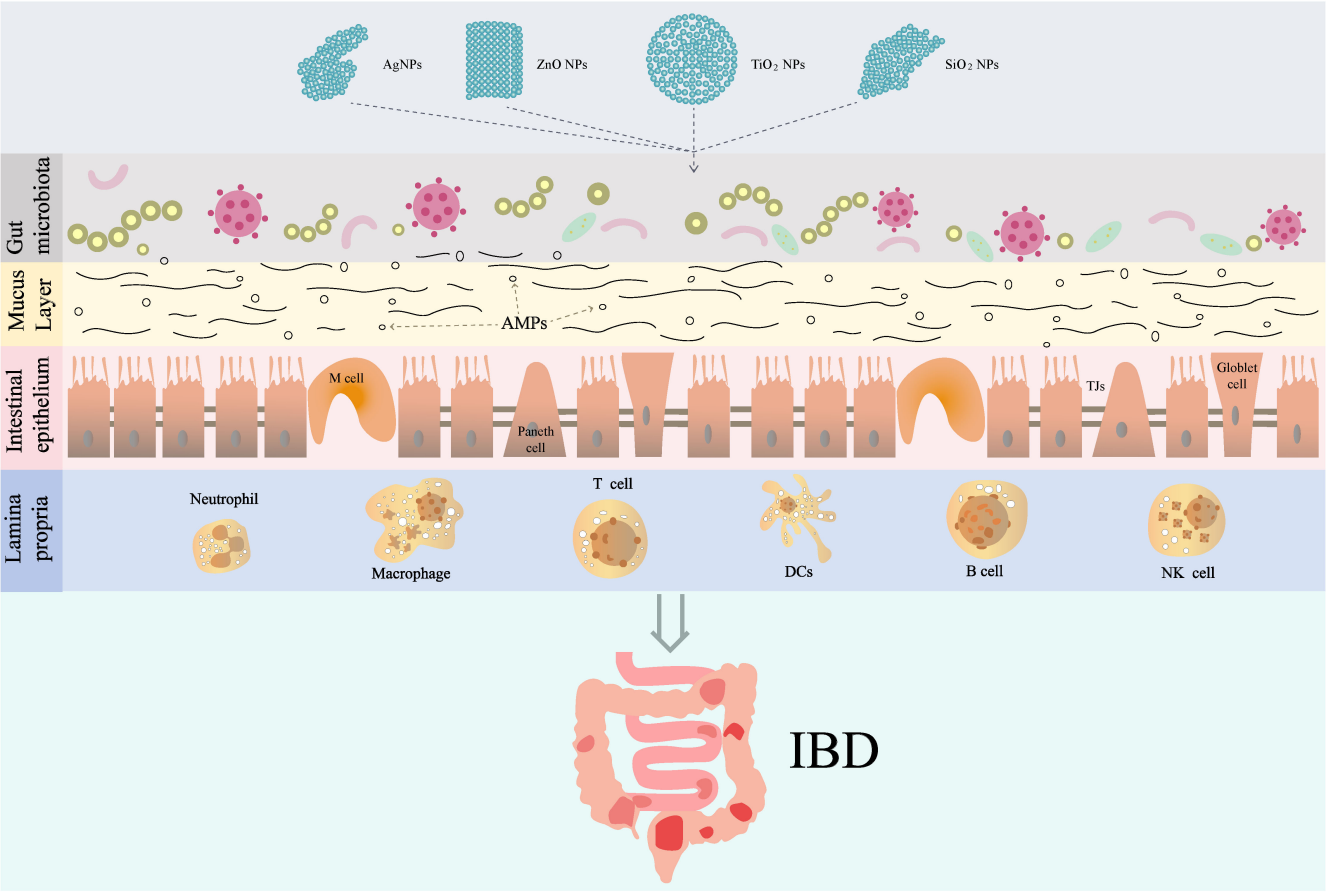
The role and mechanism of IDNPs in IBD. TJs, Tight junctions; AMPs, antimicrobial peptides; DCs, dendritic cells.

The pathogenesis of IBD is multifactorial, involving environmental factors, dysregulation of the immune response, alterations in the gut microbiota, and genetic predispositions (49). The intestinal barrier is a complex and dynamic structure that mainly includes physical barrier, biological barrier, chemical barrier, and immune barrier, playing a crucial role in maintaining homeostasis within the gastrointestinal tract (**Figure 2**). Intestinal barrier serves as the first line of defense against luminal antigens, pathogens, and toxins. Previous studies have shown that damage to the intestinal barrier structure and function plays an important role in the pathogenesis of IBD (50–52).

### 3.1 Physical barrier in IBD

Intestinal physical barrier is maintained by a complex interplay of epithelial cells, and tight junctions, which collectively regulate permeability and immune responses. Intestinal epithelial cells (IECs) are single-layer columnar cells that cover the surface of the intestine, mainly including absorptive epithelial cells, goblet cells, endocrine cells, and Paneth cells (53). Normal IECs are in a continuously renewing state, with intestinal stem cells generating new epithelial cells daily to replace those that shed. The dynamic equilibrium established by this turnover ensures the overall number of IECs remains constant. In diseased states, an abnormal increase in IEC mortality disrupts the structure and function of the intestinal mucosa. Gut microbiota can then exacerbate intestinal inflammation through this compromised mucosa. Conversely, abnormal inflammation can trigger the death of IECs, amplifying the inflammatory response and thus initiating a vicious cycle, from intestinal mucosal damage to severe intestinal inflammation (54, 55). Moreover, the loss of specific epithelial cell types, such as Paneth cells and goblet cells, has been associated with increased susceptibility to inflammation and apoptosis in IBD. Paneth cells play a crucial role in maintaining gut homeostasis by secreting antimicrobial peptides, and their depletion has been linked to dysbiosis and inflammation (56). Similarly, goblet cells are essential for mucus production, which protects the epithelial barrier; their loss can lead to increased epithelial permeability and inflammation (57, 58).

Tight junctions (TJs) are specialized structures formed by a complex of proteins, including claudins, occludin, and zonula occludens (ZO) proteins, which regulate paracellular permeability and maintain epithelial integrity (59, 60). Human studies have demonstrated that claudin-2 is upregulated in active Crohn’s disease, leading to increased paracellular permeability, while claudin-5 and claudin-8 are downregulated, contributing to barrier dysfunction (61, 62). In addition, other studies have reported downregulation of claudin-1, claudin-3, and claudin-4 in inflamed tissues of UC patients, which correlates with increased permeability and barrier dysfunction (63). Previous animal studies have shown that in animal models of dextran sulfate sodium (DSS)-induced colitis, there is a significant reduction in the expression of tight junction proteins such as ZO-1 and claudins, which correlates with increased intestinal permeability (64, 65). This dysregulation is not merely a consequence of inflammation but appears to precede the onset of severe bowel inflammation, indicating a potential early therapeutic target for intervention in IBD (64, 66). Furthermore, the inflammatory cytokines, particularly tumor necrosis factor-alpha (TNF-α) and interferon-gamma (IFN-γ), have been shown to disrupt tight junction integrity by altering the expression and localization of these proteins, thereby contributing to the progression of IBD (67, 68).

### 3.2 Biological barrier in IBD

The intestinal biological barrier, also known as the microecological barrier, refers to the normal microbiota in the intestine. The gut microbiota is a complex and dynamic ecosystem comprising trillions of microorganisms, including bacteria, fungi, viruses, and archaea. Gut microbiota is involved in synthesizing essential vitamins and metabolites, modulating the immune system, and protecting the host against pathogens by outcompeting harmful bacteria (69). In a healthy individual, the gut microbiota consists of a diverse array of microbial species that coexist in a balanced state, typically dominated by four main phyla: Firmicutes, Bacteroidetes, Actinobacteriota, and Proteobacteria. This diversity is crucial as it contributes to the overall stability and resilience of the microbiome, allowing it to perform essential functions such as digestion, metabolism, and immune modulation. The composition of gut microbiota can vary significantly between individuals due to factors such as genetics, diet, age, and environmental exposures. Studies have shown that a higher diversity of gut microbiota is generally associated with better health outcomes, while a decrease in diversity can lead to dysbiosis, which is linked to various health conditions, including IBD, obesity, and metabolic syndrome (70, 71). For instance, a reduction in the abundance of Firmicutes, particularly Faecalibacterium prausnitzii, has been linked to increased disease severity in IBD patients, while an overgrowth of Proteobacteria correlates with inflammation and disease flares (72, 73). This dysbiotic state not only exacerbates the local immune response but also disrupts the gut barrier function, contributing to the pathophysiology of IBD and increasing the risk of complications such as colonic cancer (74).

The bacterial metabolites short-chain fatty acids (SCFAs), primarily produced by the fermentation of dietary fibers by gut microbiota, play a pivotal role in maintaining gut health and modulating inflammation, particularly in the context of IBD. Research indicates that SCFAs, such as butyrate, propionate, and acetate, exert protective effects by enhancing the integrity of the intestinal barrier, modulating immune responses, and exhibiting anti-inflammatory properties. For instance, butyrate has been shown to promote the differentiation of regulatory T cells, which are crucial for maintaining immune homeostasis and preventing excessive inflammatory responses in the gut (75). Furthermore, SCFAs can inhibit histone deacetylases, leading to the upregulation of anti-inflammatory genes and the downregulation of pro-inflammatory cytokines, thus alleviating the inflammatory processes associated with IBD (76). Additionally, studies have demonstrated that SCFAs can enhance mucus production and strengthen tight junctions between epithelial cells, thereby improving gut barrier function and reducing permeability, which is often compromised in IBD patients (77). The therapeutic potential of SCFAs is being explored, with dietary interventions aimed at increasing SCFAs production showing promise in managing IBD symptoms and promoting remission (78).

### 3.3 Chemical barrier in IBD

The intestinal chemical barrier comprises various components, including the mucus layer, and antimicrobial peptides (AMPs) that work together to prevent harmful substances from penetrating the intestinal epithelium. The mucus layer is primarily composed of mucins, and glycoproteins secreted by goblet cells, which play a vital role in maintaining intestinal homeostasis and immune function (79–81). Goblet cells are crucial for the production of mucins, particularly MUC2, which forms the gel-like structure of the mucus layer. In IBD, the number and function of goblet cells are often impaired, leading to reduced mucin production and a thinner mucus layer (82–84). This reduction in mucus thickness allows for closer contact between luminal bacteria and the intestinal epithelium, potentially leading to increased inflammation and further mucosal damage (85, 86). Cytokines such as TNF-α and IL-6 have been implicated in the downregulation of mucin production, contributing to the deterioration of the mucus layer (87, 88).

AMPs are essential components of the intestinal chemical barrier, exhibiting broad-spectrum antimicrobial activity against bacteria, viruses, and fungi. These peptides are produced by various cells in the gut, including Paneth cells and epithelial cells, and are crucial for maintaining gut homeostasis. AMPs can disrupt microbial membranes, inhibit cell wall synthesis, and modulate immune responses, making them vital for the innate immune defense (6, 7). Different types of AMPs, such as defensins and cathelicidins, have been identified, each with unique mechanisms of action. The production of AMPs can be influenced by various factors, including microbial composition and inflammatory signals, highlighting their role in the gut’s response to pathogenic challenges (8, 9). In IBD, a decrease in the number of Paneth cells may lead to a reduction in the production of AMPs, resulting in dysbiosis of the gut microbiota and promotion of intestinal inflammation (56).

### 3.4 Immune barrier in IBD

The gut immune barrier is a complex and dynamic network that comprises various immune cells, including T cells, B cells, macrophages, NK cells, neutrophil, and dendritic cells, which are strategically located within the mucosa-associated lymphoid tissue (MALT). The MALT includes structures such as Peyer’s patches, isolated lymphoid follicles, and mesenteric lymph nodes, all of which are essential for the initiation and regulation of immune responses (89). Peyer’s patches, located in the ileum, are particularly important for sampling luminal antigens and facilitating the activation of B and T cells. They contain specialized epithelial cells called M cells that transport antigens from the gut lumen to underlying immune cells, thereby initiating immune responses (90). The presence of MALT in the gut allows for a rapid and robust immune response to pathogens while also promoting tolerance to harmless antigens, such as food proteins and commensal bacteria. Moreover, the role of MALT extends beyond the gut; it is also involved in the systemic immune response. The lymphocytes activated in the gut can migrate to other tissues, contributing to the overall immune surveillance of the body (91). Immune cells in the MALT, particularly T cells, B cells, and innate lymphoid cells, secrete a variety of cytokines that play critical roles in the pathogenesis of IBD. These cytokines mediate cell-cell communication and orchestrate the immune response. For instance, pro-inflammatory cytokines such as IL-17 and TNF-α are often elevated in IBD patients and contribute to the inflammatory processes that characterize the disease (92, 93). On the other hand, regulatory T cells (Tregs) produce anti-inflammatory cytokines like IL-10, which help to maintain immune tolerance and prevent excessive inflammation (94). The balance between pro-inflammatory and anti-inflammatory cytokines is crucial for the maintenance of intestinal homeostasis. Dysregulation of this balance can lead to chronic inflammation and tissue damage, contributing to the progression of IBD (95, 96).

## 4 The role and possible mechanisms of IDNPs in IBD

The effect of IDNPs on IBD has been studied in recent years. Riuz et al. have found that patients with active UC exhibit elevated levels of titanium in their blood compared to healthy controls and patients in remission from UC (10). And they found that TiO_2_ NPs treatment promotes intestinal inflammation in DSS-induced colitis mice via activation of NLRP3 inflammasome. However, other researchers have found that TiO_2_ NPs can alleviate 2,4,6-trinitrobenzenesulfonic acid (TNBS)-induced colitis in mice (9). Similarly, the effect of Ag NPs on IBD is also contradictory. For instance, Chen et al. have shown that Ag NPs induce colitis-like symptoms in the mucosa of the small intestine (97). Conversely, Siczek et al. have reported beneficial effects of Ag NPs on DSS-induced colitis (98). This may reflect the diversity of the effects of NPs on IBD, and the possible mechanisms by which IDNPs affect IBD are discussed as follows (**Figure 2**):

### 4.1 IDNPs on physical barrier injury

IDNPs mainly affect intestinal epithelial permeability by influencing the IECs and tight junctions adjacent to epithelial cells. Yan et al. indicate that exposure to TiO_2_ NPs leads to a significant downregulation of tight junction proteins such as occludin, and ZO-1 in the intestinal tract of juvenile mice (99). However, the expression levels of ZO-1 and claudin-2 proteins were not affected by exposure of Caco-2 cells, a widely used model for human intestinal epithelium, to SiO_2_ NPs (100). Besides, tight junction (Cldn1, Cldn5, Cldn6, Cldn10 and Pecam1) genes were all upregulated significantly in the ileum of female rats treated with 10 nm Ag NPs (101). In addition, Li et al. found that ZnO NPs increased the level of the ZO-1 and Claudin genes, and decreased expression of the Cyt-c and Caspase-3 levels in bovine intestinal epithelial cells (102). Interestingly, other studies have shown that exposure to Ag and ZnO NPs can display cytotoxicity, as evidenced by decreased levels of cell viability of Caco-2 cell (103, 104). This may be due to the inconsistent dosage and size of nanoparticles used in different research. (**Table 1**)

**Table 1:** The effect of different IDNPs on intestinal tight junction proteins.

IDNPs	Model	Results	Year	Reference
TiO_2_ NPs	ICR mice	Occludin, and ZO-1 significantly downregulated.	2022	(99)
SiO_2_ NPs	Caco-2 cells	ZO-1 and Claudin-2 are unaffected.	2020	(100)
Ag NPs	Sprague-Dawley rats	Cldn1, Cldn5, Cldn6, Cldn10 and Pecam1gene levels are upregulated.	2019	(101)
ZnO NPs	Bovine intestinal epithelial cells	ZO-1 and Claudin gene levels are upregulated.	2024	(102)

ICR, Institute of cancer research.

### 4.2 IDNPs on gut microbiota

#### 4.2.1 TiO_2_ NPs

Some studies suggest that TiO_2_ NPs have minimal impact on gut microbiota at low concentrations. For instance, one study utilizing a defined model intestinal bacterial community found only minor reductions in Bacteroides ovatus and an increase in Clostridium cocleatum following exposure to food-grade TiO_2_ NPs at doses relevant to humans, such as after consuming one to two pieces of gum or candy (105). Similarly, another study exposing mice to 2.5 mg/kg body weight/day of TiO_2_ NPs for 7 days found no changes in the composition of fecal microbiota (97). These findings indicate that, under certain conditions, TiO_2_ NPs may not significantly disturb gut microbial balance. Conversely, other studies have reported more pronounced effects. A study utilizing a model microbial community within a model colon observed alterations in the microbial community’ s phenotype, including significant changes in bacterial metabolites such as SCFAs, after administering 3 mg/L TiO_2_ for 5 days (106). Another investigation found that the rutile form of TiO_2_ NPs increased the abundance of Proteobacteria, while the anatase form did not; both forms significantly decreased the genus Prevotella (107). Additionally, rutile NPs increased levels of Rhodococcus, whereas anatase NPs raised levels of Bacteroides (107). These findings suggest that the chemical and physical properties of TiO_2_ NPs, including their form and coating, influence their impact on gut microbiota. Besides, considering the long-term exposure of modern humans to IDNPs, the duration of exposure seems to be a key factor in the diversity of results across different studies. The mechanisms by which TiO_2_ NPs alter gut microbiota are not fully understood but may involve several factors. The small size and large surface area of TiO_2_ NPs facilitate interaction with bacterial cells, potentially damaging cell membranes or interfering with bacterial metabolism. Additionally, TiO_2_ NPs may indirectly affect gut microbiota by altering the gut environment, such as by reducing pH or affecting nutrient availability. Chronic exposure to TiO_2_ NPs may also lead to cumulative effects on gut microbiota, exacerbating physiological alterations induced by other factors, such as an unbalanced diet.

#### 4.2.2 SiO_2_ NPs

Chen et al. have observed an increase in microbial diversity and richness, accompanied by an enrichment of Firmicutes and Proteobacteria, while populations of Bacteroidetes and Lactobacillus decreased in mice exposed to SiO_2_ NPs at a dose relevant to human consumption for 1 week (97). This unexpected effect underscores the need for thorough risk assessment, particularly considering the low absorption rate of SiO_2_ in the human gastrointestinal tract, which may lead to accumulation in the gut lumen and prolonged exposure to the microbiota. Besides, another study has found that a decreased relative abundance of Actinobacteria in SiO_2_ exposed mice (108).

#### 4.2.3 ZnO NPs

Several studies have investigated the effects of ZnO NPs on gut microbiota, reporting varied outcomes based on the animal model, dosage, and duration of exposure. For instance, in piglets, exposure to ZnO NPs at 600 mg/kg for 14 days increased bacterial richness and diversity in the ileum, while these parameters decreased in the cecum and colon (109). The ileum specifically exhibited an increased abundance of Streptococcus and a decreased proportion of Lactobacillus. Conversely, in the colon, Lactobacillus abundance increased, while the populations of Oscillospira and Prevotella decreased (109). These findings suggest that ZnO NPs exert differential effects on microbiota composition along the gastrointestinal tract. Similar trends were observed in hens, where a dose-dependent decrease in bacterial community richness was noted in the ileal microbiota following exposure to ZnO NPs at doses ranging from 25 to 100 mg/kg for nine weeks (110). This decrease was accompanied by an increase in populations of Bacteroidetes, Fusobacteria, and Bacilli, along with a decrease in Proteobacteria and Lactobacillus (110). Besides, studies using human microbiota from healthy donors have shown that ZnO NPs can impair the production capacity of SCFAs, suggesting alterations in the metabolic activity of the gut microbiota (106). These changes in SCFAs production are significant, as SCFAs play crucial roles in maintaining gut health and providing energy to colonocytes. The mechanisms underlying the interactions between ZnO NPs and gut microbiota are not fully understood but likely involve multiple factors. The antimicrobial activity of ZnO NPs is well-documented and is thought to contribute to the observed changes in microbiota composition (106). Additionally, factors such as particle size, shape, coating, and dosage of ZnO NPs may influence their reactivity with gut bacteria and, therefore, their impact on microbiota. Therefore, ZnO NPs have the potential to cause significant alterations in both the composition and metabolic activity of the intestinal microbiota. These changes depend on various factors, including dosage, duration of exposure, and the specific region of the gastrointestinal tract.

#### 4.2.4 Ag NPs

Various studies have explored the impact of Ag NPs on the intestinal microbiota, yielding contrasting results. For example, some studies have reported non-significant changes in the cecal microbiota composition of mice and rats following oral exposure to Ag NPs, regardless of particle size (111, 112). In contrast, other studies have documented shifts in microbial populations, characterized by increased proportions of Bacteroidetes and pathogenic gram-negative bacteria, alongside decreased proportions of Firmicutes, Lactobacillus, and Bifidobacterium (113). These alterations were sex-dependent, with males generally exhibiting more prominent changes in Lactobacillus and Bacteroidetes, while females showed a higher increase in Enterobacteria (113). This sex-dependency could be attributed to physiological differences in the gut microbiome between males and females, as well as variations in NPs biodistribution and toxicity. Additionally, the size of Ag NPs has been implicated as an influencing factor, albeit with heterogeneous results that do not conclusively determine a definitive size or dose effect (97, 111–114). Despite the abundance of rodent studies, data on the effects of Ag NPs on human gut microbiota are limited. One *in vitro* study determined the short-term impacts of Ag NPs on a defined human bacterial community, observing alterations in bacterial composition characterized by decreased abundances of beneficial bacteria and increased proportions of pathogenic species (115). These findings suggest that Ag NPs may similarly disrupt the balance of human gut microbiota, albeit the extent and consequences of these alterations require further investigation.

Mechanistically, Ag NPs exert their antimicrobial effects through various pathways. One proposed mechanism involves the release of toxic Ag ions, which are responsible for the antimicrobial activity of Ag NPs. However, sulfidation of Ag NPs, which can occur in the presence of sulfur-containing food matrices, limits the release of these ions and may contribute to the discrepancies observed in rodent studies (116). Additionally, the thinner cell membranes of gram-negative bacteria render them more vulnerable to Ag NPs toxicity, as evidenced by reduced abundances of gram-negative anaerobes such as Bacteroides ovatus after Ag NPs treatment (114). Besides, studies *in vitro* have shown that Ag NPs can alter the metabolic activity of microbes, resulting in reduced gas production and changes in fatty acid profiles (114, 115). These alterations suggest that Ag NPs impact the functional aspects of the gut microbiota, potentially affecting nutrient metabolism and energy homeostasis. Therefore, Ag NPs exhibit complex and multifaceted effects on the gut microbiota, influenced by factors such as dose, size, duration of exposure, and sex of the host. While some studies report profound alterations in microbial populations, others suggest minimal impact. The mechanisms underlying these effects are not fully understood but likely involve the release of toxic ions, disruption of microbial metabolism, and sex-dependent differences in NPs biodistribution and toxicity.

### 4.3 IDNPs on chemical barrier

To date, no studies have investigated the effects of IDNPs on antimicrobial peptides. Various IDNPs have effects on the intestinal mucus layer. Limage et al. demonstrated that exposure to TiO_2_ NPs resulted in changes to the mucus layer’s thickness and composition, which could have profound implications for gut health and microbiota interactions (117). Besides, Jeong et al. found that rats exposed to Ag NPs (60 nm) for 28 days exhibited increased mucus secretion, and the levels of neutral and acidic mucins in the goblet cells of the ileum, colon, or rectum were significantly decreased (118). Among the acidic mucins, the proportion of sulfated mucins declined, while the proportion of sialylated mucins increased (118). Van den Brule et al. reported that goblet cells in the ileum of Ag NPs-treated mice were not significantly affected, and the integrity of the glycocalyx was maintained (116). Williams et al. found that the administration of Ag NPs to male and female rats had little effect on MUC2 expression but induced a decrease in MUC3 expression in the ileum. The reduction of MUC3 in female rats was more significant compared to male rats (113). Although there are no studies on the effects of ZnO NPs on the intestinal mucus layer, previous research has shown that zinc deficiency can disrupt mucus production (119). Given the complex interactions between nanoparticles and the intestinal mucus layer, further experiments are needed to verify the impact of ZnO NPs.

### 4.4 IDNPs on immune barrier

#### 4.4.1 TiO_2_ NPs

Previous study has demonstrated that TiO_2_ NPs can traverse the ileum epithelium and Peyer’ s patches, leading to epithelial impairment and chronic damage (32). This initial interaction paves the way for subsequent immune disturbances. Upon entering the intestinal mucosa, TiO_2_ NPs disrupt tight junctions between intestinal epithelial cells, compromising the intestinal barrier function (99). This disruption not only facilitates the translocation of additional particles and microbial components but also initiates an inflammatory cascade (99). *In vitro* study further corroborate these findings, revealing that TiO_2_ NPs induce the expression of pro-inflammatory cytokines such as TNF-α, IFN-γ, and IL-12, which are hallmarks of a Th1-mediated immune response (120). *In vivo* studies using animal models have also highlighted the immune-modulatory effects of TiO_2_ NPs. Exposure to TiO_2_ NPs has been associated with increased production of both Th1 and Th2 cytokines in the mucosa of the small intestine, indicating a complex interplay between different immune pathways (121, 122). Notably, these immune disturbances coincide with microbiota dysbiosis, further perpetuating the inflammatory state (121). The mechanisms underlying these immune effects are multifaceted. TiO_2_ NPs have been shown to prime macrophages with an abnormal activation state, characterized by an excessive pro-inflammatory phenotype and suppressed innate immune function (123). Additionally, TiO_2_ NPs induce mitochondrial dysfunction and oxidative stress, which can exacerbate inflammation and impair immune cell function (123). Of particular concern is the potential for TiO_2_ NPs to worsen pre-existing intestinal inflammation, such as that observed in IBD (10). Studies in animal models of IBD have demonstrated that oral administration of TiO_2_ NPs exacerbates intestinal inflammation through the activation of the NLRP3 inflammasome, resulting in the release of pro-inflammatory cytokines IL-1β and IL-18 (10). These findings align with clinical observations of increased blood titanium levels and TiO_2_ particle accumulation in the Peyer’ s patches of IBD patients (10). Moreover, TiO_2_ NPs have been implicated in the development of colitis-associated cancer (CAC). Chronic inflammatory states, such as those induced by TiO_2_ NPs, predispose individuals to colorectal cancer (CRC) (24, 124). By disrupting the intestinal barrier and promoting a pro-inflammatory microenvironment, TiO_2_ NPs may facilitate the progression from inflammation to neoplasia (24, 124). Therefore, TiO_2_ NPs exert profound effects on the intestinal immune system through multiple mechanisms, including disruption of the intestinal barrier, induction of pro-inflammatory cytokines, and modulation of macrophage function. These immune disturbances are accompanied by microbiota dysbiosis, further perpetuating the inflammatory state and potentially increasing the risk of chronic diseases such as IBD and CRC.

#### 4.4.2 SiO_2_ NPs

The impact of SiO_2_ NPs on intestinal immunity is multifaceted. A study has demonstrated that SiO_2_ NPs can elicit a pro-inflammatory response in the gut. For instance, oral administration of SiO_2_ NPs to mice has been shown to increase the production of pro-inflammatory cytokines in the small intestine and colon, accompanied by histological evidence of epithelial damage and crypt loss (125). This finding suggests that SiO_2_ NPs may disrupt the intestinal barrier, leading to the translocation of bacteria and other harmful substances and thereby triggering an immune response. However, not all studies have reported adverse effects of SiO_2_ NPs on gut immunity. Other research has found no significant changes in hematological, histopathological, or biochemical properties in rats and mice orally administered SiO_2_ NPs of varying sizes and surface properties (126). These studies suggest that the toxicity of SiO_2_ NPs may be influenced by particle characteristics such as size and surface coating, as well as by the experimental conditions and animal models employed. The mechanisms underlying the immune effects of SiO_2_ NPs are not fully understood but likely involve interactions with the gut microbiota and immune cells. SiO_2_ NPs may disrupt the balance of gut microbiota, leading to dysbiosis and an altered immune response. Additionally, these nanoparticles may directly interact with immune cells, such as macrophages and dendritic cells, modulating their function and affecting the production of cytokines and other immune mediators. Therefore, the effects of SiO_2_ NPs on gut immunity are complex and contingent on multiple factors. While some studies have reported adverse effects, such as pro-inflammatory responses and epithelial damage, others have found no significant changes.

#### 4.4.3 ZnO NPs

Existing research indicates that zinc oxide nanoparticles (ZnO-NPs) have a protect effects on intestinal immunity. For instance, in piglets, ZnO-NPs have been shown to enhance the expression of antioxidant enzymes and tight junction proteins, both of which are crucial for maintaining intestinal barrier integrity (109). These effects are believed to reduce stress associated with weaning in piglets by mitigating oxidative damage and improving gut barrier function (109). Furthermore, ZnO-NPs have been reported to promote proliferation and inhibit apoptosis in enterocytes, indicating a potential role in intestinal tissue repair and regeneration. These findings are supported by studies demonstrating that ZnO-NPs derived from plant extracts exhibit antioxidant and anti-inflammatory properties, suggesting their potential as therapeutic agents for mitigating gut inflammation (127). The mechanisms underlying the effects of ZnO-NPs on intestinal immunity are multifaceted. ZnO-NPs appear to modulate the production of cytokines, which are critical for regulating immune responses. For instance, ZnO-NPs have been reported to decrease the expression of pro-inflammatory cytokines in rat models of colitis, suggesting an anti-inflammatory effect (109, 128). Xia et al. found that the expression levels of pro-inflammatory cytokines IFN - γ, IL-1 β, TNF - α, and NF - κ B were reduced in the ileum after treatment with ZnO-NPs (109). Similarly, Li et al. found that ZnO-NPs can reduce the expression of pro-inflammatory cytokines IL-1 β and TNF - α in DSS treated mice, which may be related to the activation of the Nrf2 pathway (128).

#### 4.4.4 Ag NPs

Numerous studies have investigated the interaction of Ag NPs with the gastrointestinal tract, revealing their potential to modulate both innate and adaptive immune responses. For instance, a study has shown that Ag NPs induce colitis-like symptoms characterized by increased intestinal epithelial microvilli disruption, elevated histological scores, and the production of pro-inflammatory cytokines such as TNF-α, IFN-γ, and IL-4 in the mucosa of the small intestine (97). This finding indicates that a pro-inflammatory mechanism by which Ag NPs may exacerbate intestinal inflammation and disrupt immune homeostasis (97). Conversely, another research has reported beneficial effects of Ag NPs on gastrointestinal tract health. For example, in a mouse model of DSS-induced colitis, Ag NPs significantly decreased the macroscopic score and effectively attenuated colonic damage (98). These results indicate that Ag NPs may exhibit anti-inflammatory properties under certain conditions, which could be harnessed for therapeutic purposes in the treatment of IBD. The contrasting effects of Ag NPs on gut immunity may stem from differences in particle characteristics, such as size, shape, surface coating, and aggregation state, all of which can influence biodistribution, cellular uptake, and toxicity. Additionally, gut microbiota plays a crucial role in modulating the immune response to Ag NPs, with variations in microbiota composition among individuals potentially contributing to divergent responses (111, 112). Therefore, the effects of Ag NPs on gut immunity are multifaceted and depend on various factors.

## 5 Current challenges and dilemmas

The Western diet, characterized by high fat, low fiber, and high sugar intake, has been implicated in the rising incidence of IBD. And Western diet is accompanied by the consumption of various food additives, which contains different IDNPs. In recent years, numerous studies have examined the role and mechanisms of IDNPs in IBD; however, a definitive conclusion has not yet been reached. The primary challenges we currently face include the following: First, due to variations in nanoparticles and animal models, conclusions drawn from different studies are often inconsistent or even contradictory. This inconsistency may arise from differences in the source, size, shape, and dosage of nanoparticles used across research institutions, as well as variations in the type, sex, age and dietary habits (including proportions of protein, lipid, and carbohydrate) of the animals. These factors influence the absorption, function, and metabolism of nanoparticles in the body, ultimately leading to deviations in research outcomes. Given that the human body is a complex biological system, with variations in diet, pre-existing conditions, medication use, and disease activity among different patients with IBD, the impact of INDPs on individuals may be more intricate. Second, previous studies in animals and humans often focus on the short-term effects of IDNPs exposure, while research on chronic toxicity resulting from long-term exposure *in vivo* remains limited. Since the impact of IDNPs on populations is likely to be prolonged and continuous, this issue warrants greater attention. Third, the consumption levels of IDNPs can vary significantly among individuals of differing cultural backgrounds, dietary habits, and ages; however, there is currently no reliable and accurate method for calculating intake. This presents a challenge for animal research, as imprecise dosage calculations can lead to inaccuracies when extrapolating results to human populations. Forth, considering that food packaging does not directly come into contact with the human body in practical scenarios (humans do not consume the outer packaging), there is currently no relevant research on the exposure levels and effects of IDNPs in food packaging. However, this often-overlooked issue may also impact IBD, and future research should focus on this area. Finally, a unified standard for the toxicity monitoring of IDNPs remains lacking, representing a significant gap in disease controlling. Given the widespread use of food additives, this presents a serious challenge for human health.

## 6 Directions for future exploration

To address the aforementioned challenges, further research is needed to investigate the pathophysiological changes associated with nanoparticles from different sources in various human environments. For patients with IBD, the intake of IDNPs should be approached with caution, taking into account individual characteristics. After resolving these issues, IDNPs could play a more significant role in the diagnosis and treatment of IBD. For example, an IBD disease activity risk model or prognosis model could be established based on varying blood concentrations of IDNPs, enabling more accurate predictions for disease diagnosis and prognosis. There should also be some clinical studies on the effects of IDNPs in food additives on the disease activity of IBD to obtain more accurate conclusions. In terms of treatment, consuming foods containing IDNPs that have therapeutic effects may benefit patients’ conditions. Additionally, modifying IDNPs and researching drug-loaded IDNPs may represent future directions for IBD treatment.

## 7 Conclusion

IDNPs commonly used in food additives and packaging, pose a significant yet often neglected risk for patients with IBD. This review examines their impact and mechanisms on the intestinal barrier in IBD. Previous studies have demonstrated that different types of nanoparticles exert varying effects on animals in different states. In this context, factors such as the NPs’ source, size, shape, dosage, and duration of action, as well as the animal’ s species, gender, dietary habits, and age, significantly influence the research results. Future research should explore the effects and mechanisms of diverse IDNPs on patients with IBD in greater depth.
